# Supplementary material for: Netcombin: An algorithm for constructing optimal phylogenetic network from rooted triplets
Source: PLoS One. 2020 Sep 18;15(9):e0227842. doi: 10.1371/journal.pone.0227842 (PMC7500971; doi:10.1371/journal.pone.0227842)
Supplement: S4 File — (ZIP) [file pone.0227842.s004.zip › SequenceData-Triplet/sequenceData-10-20-30-0-Triplet/length 400, numseq 30, rate 10^-7/3 seq.pdf]

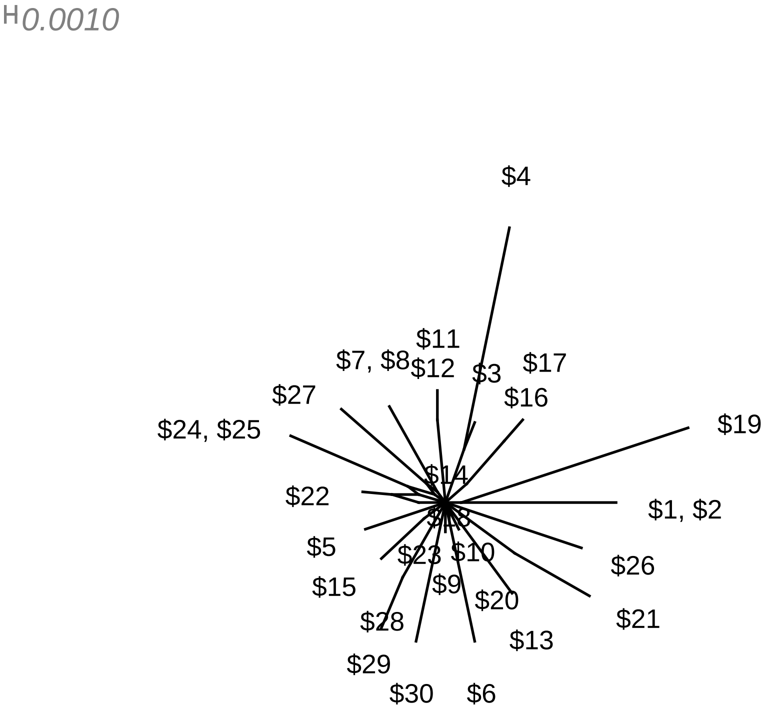

0.0010
$4
$11
$7, $8
$12
$3
$17
$27
$16
$24, $25
$19
$14
$22
$1, $2
$18
$5
$23
$10
$26
$15
$9
$20
$21
$28
$13
$29
$30
$6
